# Bioactive Properties and Chemical Composition of Wild Edible Species

**DOI:** 10.3390/molecules29133226

**Published:** 2024-07-08

**Authors:** Spyridon A. Petropoulos

**Affiliations:** Laboratory of Vegetable Production, University of Thessaly, Fytokou Street, 38446 Volos, Greece; spetropoulos@uth.gr

## 1. Introduction

Wild edible species are usually collected from the wild, and they have been included in the human diet beyond the advent of agriculture, as confirmed by several ethnobotanical surveys [1,2,3]. They are commonly used in a broad range of local and traditional dishes and are highly appreciated for their distinct taste and aroma, which complement the quality and health benefits of food products [4]. Their unique properties are associated with phytochemical compounds, including various polyphenols, vitamins, macro- and microminerals, and several antioxidant compounds [5,6]. In particular, polyphenols are the most well-known phytochemicals, with several studies to prove their prominent bioactive properties and their contribution to health improvement [7]. Although they are not considered essential nutrients in human diets, their inclusion in a daily diet is accompanied by several beneficial health effects [2,8]. They can be found in numerous species throughout the plant kingdom, as they play a major role in the defense and protection of plants against various stressors, including biotic and abiotic ones [9].

Many of these species are key ingredients in several diets around the world, and despite being used in low amounts, they can contribute to the improvement of the health and well-being of the general public through the prevention of serious chronic diseases associated with modern lifestyles (e.g., lack of exercise, fast food, oxidative stress due to anxiety, etc.) [10]. There is a great variation in the chemical profile of wild edible species that is related not only to differences in the genetic background but also to differences in growing conditions, which may significantly affect the biosynthesis of bioactive compounds and result in several ecotypes of the same species [11]. Apart from polyphenols (e.g., phenolic acids, flavonoids, isoflavonoids, anthocyanins, and lignans), several other phytochemicals may be included, such as phytoestrogens, terpenoids, carotenoids, limonoids, phytosterols, and glucosinolates, which also possess bioactive properties and can provide beneficial health effects [10].

Finally, wild edible species also contain several other important compounds, including fatty acids, free sugars, tocopherols, organic acids, and many vitamins, which are important in balanced and health-beneficial diets [10]. 

This Special Issue focuses on presenting the most up-to-date research regarding the chemical and bioactive profiles of wild and/or underutilized edible species, aiming to reveal the association between bioactive properties and the composition of phytochemicals, as well as identify those factors that regulate the biosynthesis of bioactive compounds. Moreover, this Special Issue intends to present research data regarding the effect of pre- and post-harvest factors on phytochemical composition, their bioavailability, and their potential valorization in the pharmaceutical and nutraceutical sectors and/or in the food industry, through the design of novel functional foods. 

## 2. An Overview of Published Articles

This compilation of published articles covers a wide range of wild edible species from various parts of the world and presents their chemical compositions and functional properties.

Villagrán et al. (Contribution 1) performed a literature review about an edible fungus (e.g., huitlacoche; *Ustilago maydis*), which is commonly used in Mexico as a food source. This fungus attacks maize plants and can cause severe economic losses in this important crop. However, despite its phytopathogenic activity, it can be a nutritious food product, which is highly appreciated in the domestic Mexican market due to its bioactive properties, including antioxidant, antimicrobial, anti-inflammatory, antimutagenic, antiplatelet, and dopaminergic effects. Apart from these effects, huitlacoche is also used in the industrial sector as a stabilizing and capping agent for the synthesis of inorganic nanoparticles, for the removal of heavy metals from aqueous media, as a biocontrol agent in wine production, as a biosurfactant agent, and for several other industrial applications, including the food industry sector and the design of novel functional foods. In their review, the authors also present the current status of the species as well as the strategies that have to be adopted for its further valorization and exploitation.

In the study by Urbonaviciene et al. (Contribution 2), the nutritional and physicochemical profile of wild lingonberry (*Vaccinium vitis-idaea* L.) was evaluated. The authors studied the effect that the geographic origin of a plant may have on the physicochemical and nutritional properties of lingonberry fruits, and for this purpose, they evaluated samples collected from four different regions in Northern Europe (e.g., Norway, Finland, Latvia, and Lithuania). The results of this work revealed a significant impact of growing conditions on the morphological and chemical aspects of wild lingonberry fruit, thus suggesting the importance of fastidious screening of local ecotypes for the valorization of this valuable natural source of bioactive compounds.

Silva et al. (Contribution 3) evaluated the bioactive compounds and antioxidant and cytotoxic properties of *Hibiscus acetosella* Welw. Ex Hiern (also known as vinagreira-roxa, vinagreira, groselheira (gooseberry bush), rosela, quiabo azedo (sour okra), and quiabo roxo (purple okra)), a wild species native to Africa, which is commonly used in Brazil for its edible flowers, as a green vegetable, or in decoctions obtained from the leaves and buds. According to the results of this work, the flowers are rich in organic acids and phenolic compounds (e.g., myricetin and quercetin derivatives, kaempferol, and anthocyanins), and the flower extracts did not show cytotoxic effects against the African green monkey kidney epithelial Vero, liver epithelial-like HEPG2, human kidney embryo HEK-293, mouse macrophage RAW 264.7, or the rat myoblast L6 cell lines tested, thus suggesting their safe consumption in human diets. 

Craine et al. (Contribution 4) evaluated the potential safety of Perennial Baki^TM^ beans for human consumption due to the increasing interest of consumers for novel food sources. Baki™ beans refer to perennial legumes that are obtained from *Onobrychis* spp. (also known as sainfoins), with distinct quality features. After significant domestication efforts by the research team, this study provided important preliminary results regarding the safety of Baki™ bean for human consumption based on their level of mycotoxins, heavy metals, microorganisms, and pesticides, as well as macronutrient content. The obtained results did not reveal any issues that would affect the health and safety of these products, and they also suggest a high potential for introducing these beans as a novel pulse crop suitable for human consumption. 

Duarte-Casar et al. (Contribution 5) performed a literature review regarding the bioactive properties of five wild Ecuadorian fruit: borojó (*Alibertia patinoi*); chonta (*Bactris gasipaes*); arazá (*Eugenia stipitata*); Amazon grape (*Pourouma cecropiifolia*), and cocona (*Solanum sessiliflorum*). In particular, they studied the potential uses of these species against the metabolic syndrome, and they highlighted the high economic, cultural, and nutritional value of these fruits that could be integrated into modern diets and contribute to sustainable agriculture, cultural preservation, and the health of the general public through the design of novel functional foods and nutraceuticals.

## 3. Conclusions

The compiled research and literature review papers highlight the importance of wild edible species in human diets and their contribution to the improvement of health and protection against chronic diseases. However, considering the vast number of such species and the great variability in the chemical profile and phytochemical content due to growing conditions and the genetic background, more research is needed in order to reveal the wealth of activities and further valorize/explore these species through breeding programs, with the aim to integrate them into commercial cropping systems. 
